# The hidden depths of zebrafish skin

**DOI:** 10.7554/eLife.88597

**Published:** 2023-05-23

**Authors:** Yue Rong Tan, Megan Liaw, Chen-Hui Chen

**Affiliations:** 1 Institute of Cellular and Organismic Biology (ICOB) at Academia Sinica Taipei City Taiwan

**Keywords:** development, single-cell transcriptomic, enamel, skin, ameloblasts, Zebrafish

## Abstract

Single-cell transcriptome analysis of zebrafish cells clarifies the signalling pathways controlling skin formation and reveals that some cells produce proteins required for human teeth to acquire their enamel.

**Related research article** Aman AJ, Saunders LM, Carr AA, Srivatsan SR, Eberhard CD, Carrington B, Watkins-Chow D, Pavan WJ, Trapnell C, Parichy DM. 2023. Transcriptomic profiling of tissue environments critical for post-embryonic patterning and morphogenesis of zebrafish skin. *eLife* **12**:RP86670. doi: 10.7554/eLife.86670.

The largest organ in the vertebrate body, the skin, performs a wide range of roles such as protecting against infection, sensing the environment, and supporting essential appendages such as hair, feathers and scales. It is also beautifully complex.

In its postembryonic form, vertebrate skin is formed of three layers – the epidermis (the outermost layer), the dermis and the hypodermis – that contain a range of different cell types, each dedicated to a specific function. In zebrafish, for example, some cells create the proteins required for scales to harden and become calcified, while others produce the pigments that give the species its delicate stripe pattern. Despite extensive studies over the past few decades, researchers still do not fully understand how this complexity arises during development. Now, in eLife, David Parichy and colleagues – including Andrew Aman and Lauren Saunders as joint first authors – report that they have classified all the major cell types in zebrafish skin, identified a cell type which was previously unknown, and dissected some of the signalling networks that are essential for development (Aman et al., 2023).

The researchers – who are based at the University of Virginia, the University of Washington and the National Human Genome Research Institute – started by using single-cell transcriptomic analysis to study 35,114 post-embryonic zebrafish skin cells. This approach allowed Aman et al. to establish the ‘RNA profile’ of each individual cell, showing which genes it expresses, and at what level, at a given time.

One of the most interesting findings to emerge from this work was the identification of a group of epidermal cells which expressed genes coding for proteins that are necessary for the formation of enamel (Figure 1). As human cells known as ameloblasts secrete some of the same proteins to create the enamel of our teeth, this result suggests that zebrafish scales could be an alternative model in which to study ameloblast biology in vivo. Meanwhile, it also highlights an ancient connection between fish scales and human teeth, one that may date back 450 million years to the time when the first fish species with calcified outer layers emerged during the Ordovician Period (Sire et al., 2009). In fact, some evidence suggests that teeth may have evolved from certain types of primitive scales (Gillis et al., 2017).

**Figure 1.**
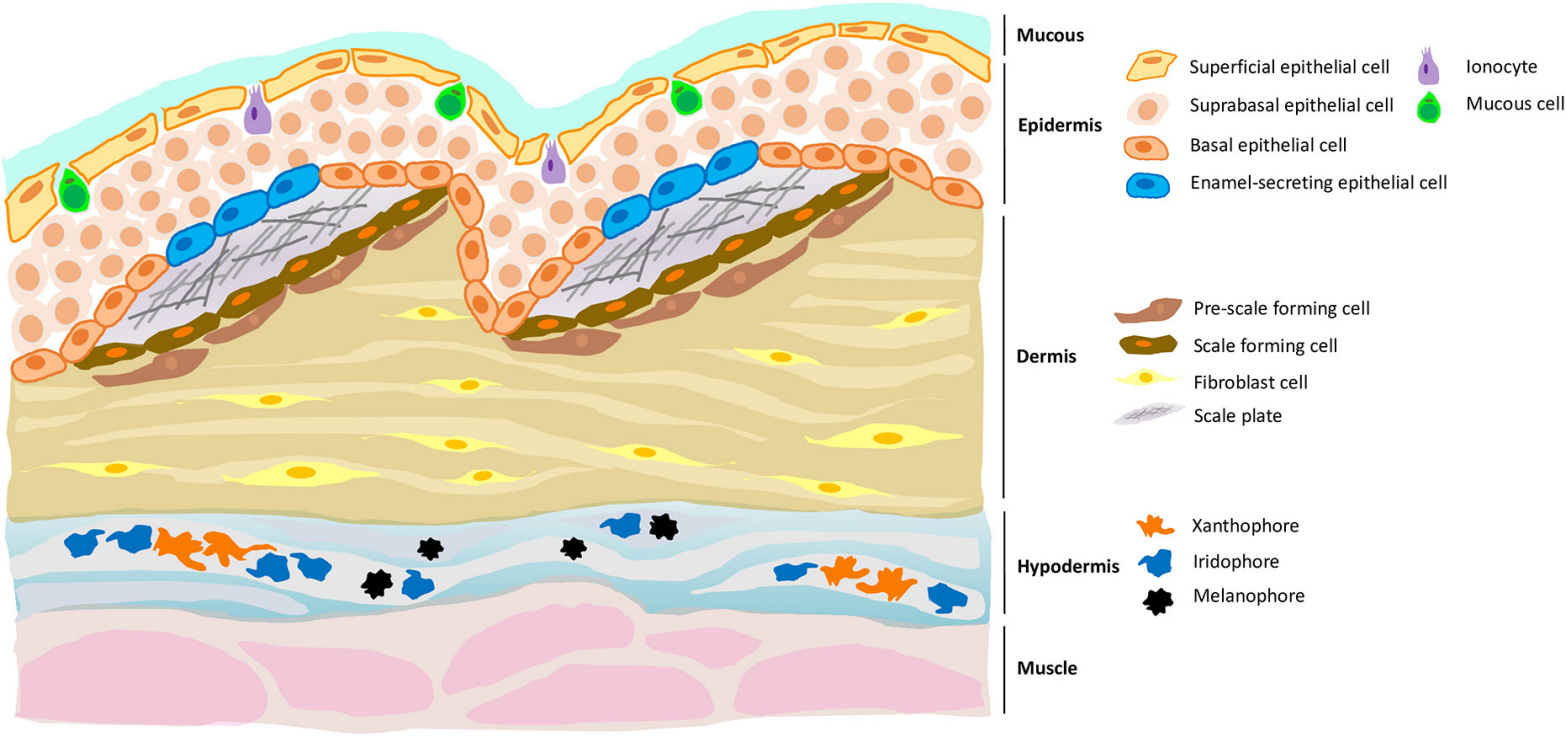
A new cell type in the epidermis of zebrafish, and a new role for the hypodermis in pigmentation. Zebrafish skin is composed of three layers, each of which contains distinct cell types. For example, the dermis (the middle layer) contains fibroblast cells, pre-scale forming cells and scale forming cells; the latter two cell types support the growth of scale plates which, when coated with a matrix that allows calcification, will become scales. Aman et al. demonstrate the presence of a previously unknown cell type (blue) in the epidermis (the top layer) which expressed genes necessary for enamel formation. Aman et al. also confirm that the hypodermis (the bottom layer) is important for pigment production, being enriched with different types of pigment cells such as xanthophores, melanophores and iridophores.

Image credit: Yue Rong Tan (CC BY 4.0).

To better understand the molecular mechanisms underpinning skin development, Aman et al. applied their approach to cells from various zebrafish mutants (Harris et al., 2008; Lang et al., 2009; McMenamin et al., 2014). In animals with scale defects, the analyses revealed several signalling pathways that act in turn to regulate scale-forming cells at the base of the epidermis. Further in vivo experiments helped to pinpoint key molecular actors in this process, highlighting a specific signalling ligand called Fgf20a, which is also involved in the development and regeneration of scales. Piecing together the RNA profiles of zebrafish mutants with defective pigment development, on the other hand, provided convincing evidence that the hypodermis is not in fact a mere structural layer. Instead, it is essential for pigment cell development and adult stripe pattern formation.

Finally, Aman et al. examined the role of the thyroid hormone on skin development, as this chemical messenger has been implicated in a range of human skin conditions. To do so, they examined the RNA profiles of skin cells from zebrafish in which the thyroid gland had been removed (McMenamin et al., 2014). This analysis revealed several genes whose expression is potentially regulated by this hormone, including a gene called *pdgfaa*. Further in vivo work showed that over-expressing this gene in fish with low levels of thyroid hormone partially re-established a normal stratification of the dermis, but did not alter how scales were created. Together, these findings should open new opportunities for understanding and treating human skin diseases.

This work illustrates how single-cell transcriptomic profiling can detect rare cell types, infer cell fate trajectories, and identify relevant signalling networks. On its own, however, this method may fall short of capturing the exquisite details of skin development, such as how differentiated skin cells influence the behavior of neighbouring basal stem cells, the way that appendages instruct the growth of nerve projections and blood vessels, or the fact that tension can trigger skin cells to divide without replicating their DNA (Mesa et al., 2018; Ning et al., 2021; Rasmussen et al., 2018; Chan et al., 2022). Only studies in live animals can investigate the role of these cell-to-cell interactions and dynamics in skin development, emphasising a need for multifaceted approaches.

Zebrafish skin may seem less sophisticated than ours at first glance, but Aman et al. have undoubtedly demonstrated that there is much to discover beneath its surface. Developmental biologists can glean valuable insights from looking into it more closely. Given the evolutionary connection between teeth and scales, and now the shared presence of ameloblast-like cells in zebrafish and humans, it may even become possible to unravel why scales, but not human teeth, can regrow throughout life. While it is probably a wild guess, it is fascinating to imagine that one day we may be able to regenerate human teeth thanks to findings made in a toothless little fish.
